# Population structure, allelic variation at *Rht-B1* and *Ppd-A1* loci and its effects on agronomic traits in Argentinian durum wheat

**DOI:** 10.1038/s41598-022-13563-w

**Published:** 2022-06-10

**Authors:** Ana Laura Achilli, Pablo Federico Roncallo, Adelina Olga Larsen, Susanne Dreisigacker, Viviana Echenique

**Affiliations:** 1 Centro de Recursos Naturales Renovables de la Zona Semiárida (CERZOS), Departamento de Agronomía, Universidad Nacional del Sur (UNS)-CONICET, Bahía Blanca, Argentina; 2 Compañía Molinera del Sur, Bahía Blanca, Argentina; 3 Ex CEI Barrow, Instituto Nacional de Tecnología Agropecuaria (INTA), Tres Arroyos and CERZOS-UNS/CONICET, Bahía Blanca, Argentina; 4 International Maize and Wheat Improvement Center (CIMMYT), Mexico, DF Mexico

**Keywords:** Plant breeding, Genetic markers, Quantitative trait

## Abstract

Exploring the genetic variability in yield and yield-related traits is essential to continue improving genetic gains. Fifty-nine Argentinian durum wheat cultivars were analyzed for important agronomic traits in three field experiments. The collection was genotyped with 3565 genome-wide SNPs and functional markers in order to determine the allelic variation at *Rht-B1* and *Ppd-A1* genes. Population structure analyses revealed the presence of three main groups, composed by old, modern and genotypes with European or CIMMYT ancestry. The photoperiod sensitivity *Ppd-A1b* allele showed higher frequency (75%) than the insensitivity one *Ppd-A1a* (*GS105*). The semi-dwarfism *Rht-B1b* and the *Ppd-A1a* (*GS105*) alleles were associated with increases in harvest index and decreases in plant height, grain protein content and earlier heading date, although only the varieties carrying the *Rht-B1* variants showed differences in grain yield. Out of the two main yield components, grain number per plant was affected by allelic variants at *Rht-B1* and *Ppd-A1* loci, while no differences were observed in thousand kernel weight. The increases in grain number per spike associated with *Rht-B1b* were attributed to a higher grain number per spikelet, whereas *Ppd-A1a* (*GS105*) was associated with higher grain number per spikelet, but also with lower spikelets per spike.

## Introduction

Durum wheat (*Triticum turgidum* L. var. *durum*) is the raw material for producing semolina for the pasta industry. Increasing the production of durum wheat contributes to supplying the future demand for food caused by steady growth of the world population^1^. As the arable land is a limited resource and reaching the crop yield potential is still a challenge, so exploring the genetic variation of yield-related traits in current elite germplasm is an important approach to understand whether enough variability is available to drive genetic gains.

In Argentina, durum wheat is a traditional crop that has been cultivated for more than 80 years, showing the highest level of production in the 1970s^2^. Compared with bread wheat, durum wheat has been associated with lower yield and greater adaptation to abiotic stress conditions^3,4^. However, breeding efforts reduced this yielding gap between bread and durum wheat in the last two decades^5^. Currently, more than 95% of the Argentinian durum wheat production is concentrated in the center east of the country (Buenos Aires province) (http://datosestimaciones.magyp.gob.ar/), being the southeast of Buenos Aires province the main production area. In this region, durum wheat has a great regional importance playing a central role for the national pasta industry that has been increased in recent years reaching 400,025 tons in 2018^6^. In the international ranking of pasta consuming countries, Argentina is among the top ten, with 8.8 kg per capita per year^7^.

The introduction of semi-dwarfing alleles of plant height genes (*Rht*) during the 1960s and 1970s produced the greatest increases in wheat yield during the Green Revolution^8^. Semi-dwarfism was introduced into Argentinian durum wheat genotypes during the 1970s through crosses with CIMMYT germplasm^2^ and it is found in all of the genotypes currently grown in Argentina and most of the durum wheat cultivated worldwide. The *Rht-B1* gene, located on chromosome 4B, is the main determinant of plant height in most durum wheat cultivars^9^, being the *Rht-B1b* semi-dwarfing allele the most common mutation. The *Rht-B1b* allele has been associated with high yield caused by an increased partitioning of assimilates to developing spikes^10,11^. Moreover, McClung et al.^12^ reported a pleiotropic effect of a semi-dwarfism gene that resulted in a greater number of tillers and kernels per spike, and lower grain protein content in durum wheat. Lower grain protein associated with *Rht-B1b* was also found in the early stages of seed development in bread wheat^13^.

Flowering time plays an important role in determining the environmental conditions during the grain filling period, having a significant impact on yield^14,15^. In wheat, flowering time is largely determined by vernalization (*Vrn*) and photoperiod sensitivity (*Ppd*) genes. Vernalization is the requirement of low temperatures to induce flowering, *Vrn-1* being the major gene involved in this process for both bread and durum wheat^16,17^. The dominant spring alleles of the homologous *Vrn-1* genes (*Vrn-A1* and *Vrn-B1*) are the most common variants in durum wheat^18,19^. Similar to major elite durum wheat gene pools worldwide^20^ and bread wheat cultivars in Argentina^21^, most of the Argentinian durum wheat cultivars have a spring growth habit, thus they do not have major vernalization requirements.

Wheats that are photoperiod sensitive need to be exposed to long days in order to flower. The major photoperiod sensitivity genes in wheat are the homologs of the *Ppd-1* gene^22^. The allelic variants of *Ppd-A1*, located on chromosome 2A, have the strongest effect on photoperiod sensitivity in durum wheat^23^. The two most important types of deletions within the wild-type sequence of the *Ppd-A1* gene in durum wheat were reported by Wilhelm et al.^24^ and named *GS100* and *GS105* (1027 or 1117 bp, respectively). Both of these deletions on the *Ppd-A1* gene are related to photoperiod insensitivity, thus accelerating flowering^20^. Photoperiod insensitivity increased yield up to 35% in bread wheat cultivars in the south of Europe^15^. Furthermore, early flowering is associated with a reduction in plant height, lower spike and spikelet numbers and a higher number of grains in bread wheat^15^. In durum wheat, Royo et al.^20^ reported increases in grain yield associated with photoperiod insensitivity by allowing more favorable environmental conditions during grain filling in Spain and Mexico.

A large number of SNP markers has been developed, distributed throughout the wheat genome. These markers have been shown to be efficient tools to assess the genetic diversity of wheat germplasm collections worldwide^25–27^. The study of the relationships between genetic groups and phenotypes for agronomically relevant traits is an approach that has been used in different wheat populations, allowing the characterization of the existing genetic variability^28–31^. Moreover, taking into account the effect of the main genes that affect key traits in wheat, such as plant height and heading date, may help to elucidate part of the differences between population groups.

An in-depth analysis of the genetic variation in yield and yield related traits and the study of genetic diversity are yet lacking for Argentinian durum wheat germplasm. In this study, a wide range of spring durum wheat genotypes grown in Argentina over the last 70 years was included to analyze how yield components contribute to yield formation in local environments and its relationship with the effect of major genes. This information could help breeders to define an appropriate strategy to increase yield potential. To study the population structure, a subset of genome-wide SNP markers selected from the Axiom 35 K array was used. The aims of this study were (1) to evaluate the phenotypic variation in 12 agronomic traits (2) to assess the population structure and genetic variation in semi-dwarfing (*Rht-B1*) and photoperiod sensitivity (*Ppd-A1*) genes in our collection and (3) to elucidate the effect of the clusters defined by the genetic structure and the allelic variants of major genes on agronomic traits.

## Results

### Genotypic characterization

The whole collection was genotyped with 35,143 SNPs. In this collection 52% of the SNPs were polymorphic (18,122 markers). Out of the 18,122 polymorphic markers, the SNPs with more than 10% of missing data and more than 10% of heterozygosity were removed, leaving 10,776 markers. Then, of these markers, the ones with a frequency of minor alleles (MAF) lower than 5% were removed, leaving 6462 markers. Finally, 3565 SNPs met the quality cutoff according to the scores for the probes produced by the Affymetrix software (‘Poly High Resolution’ and ‘Off-Target Variant’ categories were considered). These SNPs were widely distributed across the whole genome of durum wheat (Table S1) and so they were used in the population structure and genetic diversity analyses.

The allelic diversity in the *Rht-B1* and *Ppd-A1* genes was analyzed and two allelic variants were found on both loci (Table 1). Only five genotypes (8%) carried the *Rht-B1a* wild-type allele (tall plants), while 54 genotypes carried the *Rht-B1b* semi-dwarfing allele. The allelic characterization at the *Ppd-A1* gene revealed that 15 genotypes (25%) carried the *Ppd-A1a* (*GS105*) allele, conferring photoperiod insensitivity, whereas the remaining genotypes had the sensitive *Ppd-A1b* one. Our results showed that the *Rht-B1b* allele, associated with semi-dwarfism in wheat, was incorporated into Argentinian genotypes during the 1970s, and all the genotypes bred after 1980 carry this allele. However, the photoperiod insensitive *Ppd-A1a* (*GS105*) allele started to appear in local genotypes in 1987.

**Table 1:** Name, year of release, group assigned by STRUCTURE and the *Rht-B1* and *Ppd-A1* allelic variants of the durum wheat genotypes analyzed in this study. The year of release of genotypes was taken from Roncallo et al.^36^.

Genotype	Year	STRUCTURE group	*Rht-B1*	*Ppd-A1*	Genotype	Year	STRUCTURE group	*Rht-B1*	*Ppd-A1*
Taganrog	1934	Q1	***Rht-B1a***	*Ppd-A1b*	CBW 0105	nr	Q3	*Rht-B1b*	*Ppd-A1b*
Candeal Durumbuck	1952	Q1	***Rht-B1a***	*Ppd-A1b*	CBW 0111	nr	Q3	*Rht-B1b*	*Ppd-A1b*
Taganrog Sel. BUCK	1961	Q1	***Rht-B1a***	*Ppd-A1b*	CBW 0120	nr	Q3	*Rht-B1b*	*Ppd-A1b*
Taganrog Vilela Fideos	1961	Q1	***Rht-B1a***	*Ppd-A1b*	CBW 0141	nr	Q3	*Rht-B1b*	***GS105***
Taganrog Buck Balcarce	1980	Q1	***Rht-B1a***	*Ppd-A1b*	CBW 0200	nr	Q3	*Rht-B1b*	*Ppd-A1b*
Bonaerense Valverde	1980	Q1	*Rht-B1b*	*Ppd-A1b*	CBW 0230	nr	Q3	*Rht-B1b*	*Ppd-A1b*
BonINTA Cumenay	1995	Q1	*Rht-B1b*	*Ppd-A1b*	VF 003	nr	Q3	*Rht-B1b*	*Ppd-A1b*
Buck No6*	nr	Q1	*Rht-B1b*	*Ppd-A1b*	VF 0113	nr	Q3	*Rht-B1b*	*Ppd-A1b*
BF 1776*	nr	Q1	*Rht-B1b*	*Ppd-A1b*	VF 0136	nr	Q3	*Rht-B1b*	***GS105***
Buck Cristal	1988	Q2	*Rht-B1b*	*Ppd-A1b*	VF 0137	nr	Q3	*Rht-B1b*	***GS105***
Buck Ambar	1995	Q2	*Rht-B1b*	*Ppd-A1b*	VF 0154	nr	Q3	*Rht-B1b*	***GS105***
Buck Platino	2002	Q2	*Rht-B1b*	*Ppd-A1b*	VF 0163	nr	Q3	*Rht-B1b*	*Ppd-A1b*
Buck Granate	2010	Q2	*Rht-B1b*	*Ppd-A1b*	VF 0167	nr	Q3	*Rht-B1b*	*Ppd-A1b*
CBW 0112	nr	Q2	*Rht-B1b*	*Ppd-A1b*	B#24	nr	Q3	*Rht-B1b*	***GS105***
CBW 0153	nr	Q2	*Rht-B1b*	*Ppd-A1b*	B#25	nr	Q3	*Rht-B1b*	***GS105***
CBW 0156	nr	Q2	*Rht-B1b*	*Ppd-A1b*	ACA 2125.07	nr	Q3	*Rht-B1b*	***GS105***
VF 042	nr	Q2	*Rht-B1b*	*Ppd-A1b*	ACA 4420.08	nr	Q3	*Rht-B1b*	***GS105***
B#27	nr	Q2	*Rht-B1b*	*Ppd-A1b*	Balcarceño INTA	1974	M	*Rht-B1b*	*Ppd-A1b*
Buck Mechongue	1979	Q3	*Rht-B1b*	*Ppd-A1b*	BonINTA Facon	1997	M	*Rht-B1b*	***GS105***
Bonaerense Quilaco	1987	Q3	*Rht-B1b*	***GS105***	Buck Esmeralda	2000	M	*Rht-B1b*	*Ppd-A1b*
Buck Topacio	1997	Q3	*Rht-B1b*	*Ppd-A1b*	BonINTA Quillen	2015	M	*Rht-B1b*	*Ppd-A1b*
BonINTA Carilo	2002	Q3	*Rht-B1b*	*Ppd-A1b*	CBW 0210	nr	M	*Rht-B1b*	*Ppd-A1b*
ACA 1801F	2008	Q3	*Rht-B1b*	***GS105***	CBW 0225	nr	M	*Rht-B1b*	*Ppd-A1b*
ACA 1901 F	2009	Q3	*Rht-B1b*	***GS105***	CBW 05024	nr	M	*Rht-B1b*	*Ppd-A1b*
Buck Zafiro	2015	Q3	*Rht-B1b*	*Ppd-A1b*	CBW 05072	nr	M	*Rht-B1b*	*Ppd-A1b*
B33.1123.16-3-4-3	nr	Q3	*Rht-B1b*	*Ppd-A1b*	CBW 05081	nr	M	*Rht-B1b*	*Ppd-A1b*
CBW 0001	nr	Q3	*Rht-B1b*	*Ppd-A1b*	CBW 05082	nr	M	*Rht-B1b*	*Ppd-A1b*
CBW 0002	nr	Q3	*Rht-B1b*	***GS105***	CBW 08131	nr	M	*Rht-B1b*	***GS105***
CBW 0004	nr	Q3	*Rht-B1b*	***GS105***	CBW 09034	nr	M	*Rht-B1b*	*Ppd-A1b*
CBW 0101	nr	Q3	*Rht-B1b*	*Ppd-A1b*					

*nr* not released.

*Only two not released genotypes were developed before 1980. *GS105* deletion in *Ppd-A1* gene is also named as *Ppd-A1a* allele.

### Population structure and genetic diversity

**Figure 1:**
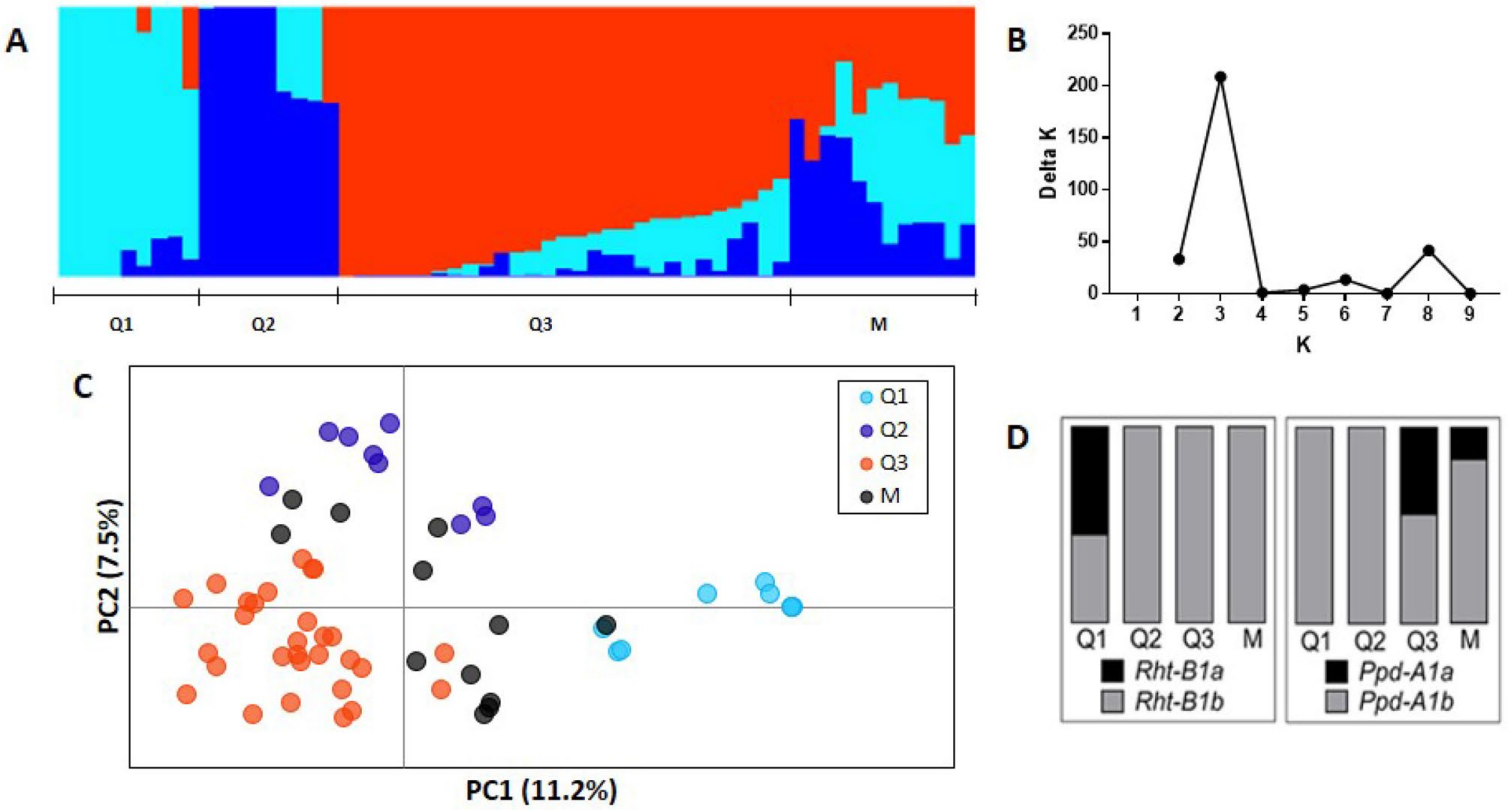
Population structure analysis for 59 Argentinian durum wheat genotypes assessed by 3565 SNP. (A) Barplot of the genotypes at K = 3 from STRUCTURE software, (B) delta K values for the different genetic groups, (C) Principal Coordinate Analysis (PCoA) and (D) *Rht-B1* and *Ppd-A1* allele frequencies in each group defined by STRUCTURE. Colors in the PCoA plot for each genotype correspond to the STRUCTURE results at K = 3. M indicates the mixed group (% of membership < 0.6), in PCoA plot the mixed genotypes were colored in black.

Population stratification was analyzed through the STRUCTURE software (Fig. 1A). The estimated ∆K suggests the presence of three genetic groups in our collection (K = 3) (Fig. 1B). Eighty percent of the genotypes were included in one of the three genetic groups, with a membership percentage higher than 60%. The remaining genotypes were considered as an admixture group (M). Principal Coordinate analysis (PCoA) explained 19% of the total genetic variation in the two first axes. The genotypes were well clustered by the PCoA in agreement with the genetic groups previously defined by the STRUCTURE analysis (Fig. 1C).

The analysis of molecular variance (AMOVA) showed that the genetic variation between the STRUCTURE groups (Q1, Q2, Q3 and M) only represented 19.7% of the total variance, whereas most of the genetic variation (80.3%) was observed within the groups (Table S2). Considering the different groups, the highest diversity values were found in Q3, the number of alleles (*Na*) being 1.95, the number of effective alleles (*Ne*) 1.52, Shannon’s information index (*I*) 0.47, unbiased expected heterozygosity (*uHe*) 0.31 and the percentage of polymorphic loci (%P) 94.5% (Table 2). On the other hand, the Q1 group was the most distant in relation to the others (mean pairwise *Fst* = 0.293) (Table 2).

**Table 2:** Diversity indices estimated for each STRUCTURE group and Pairwise Fixation index (*Fst*) between them.

	N	%P	*Na*	*Ne*	*I*	*uHe*	Q1	Q2	Q3	M
Q1	9	49.71	1.50	1.27	0.25	0.18				
Q2	9	53.13	1.53	1.30	0.28	0.19	0.392			
Q3	29	94.50	1.95	1.52	0.47	0.31	0.272	0.191		
M	12	82.41	1.82	1.49	0.43	0.30	0.216	0.154	0.086	
Total	59	100	2.00	1.54	0.49	0.33				

*N* sample size, *%P* percentage of polymorphic loci, *Na* number of alleles, *Ne* number of effective alleles, *I* Shannon’s information index, *uHe* unbiased expected heterozygosity.

The Q1 genetic group (n = 9) included most of the oldest genotypes. Eight out of nine genotypes belonging to this group were released in 1980 or earlier (Table 1) and it included all the genotypes harboring the *Rht-B1a* semi-dwarfing allele (Fig. 1D). The Q2 group included nine genotypes released by Buck Semillas SA and the Public National Breeding Program between 1988 and 2010 (Table 1). Q3 was the largest group, including 29 genotypes released between 1979 and 2015 and all the genotypes from ACA Coop. Ltda. The admixture group included 12 genotypes released between 1974 and 2015, 11 of which were developed by the Public National Breeding Program (Table 1). All the genotypes carrying the *Ppd-A1a* allele were included in Q3 or in the admixture group (45 and 17% of the genotypes in each group, respectively) (Fig. 1D).

### Environmental conditions and phenotypic traits variation

**Figure 2:**
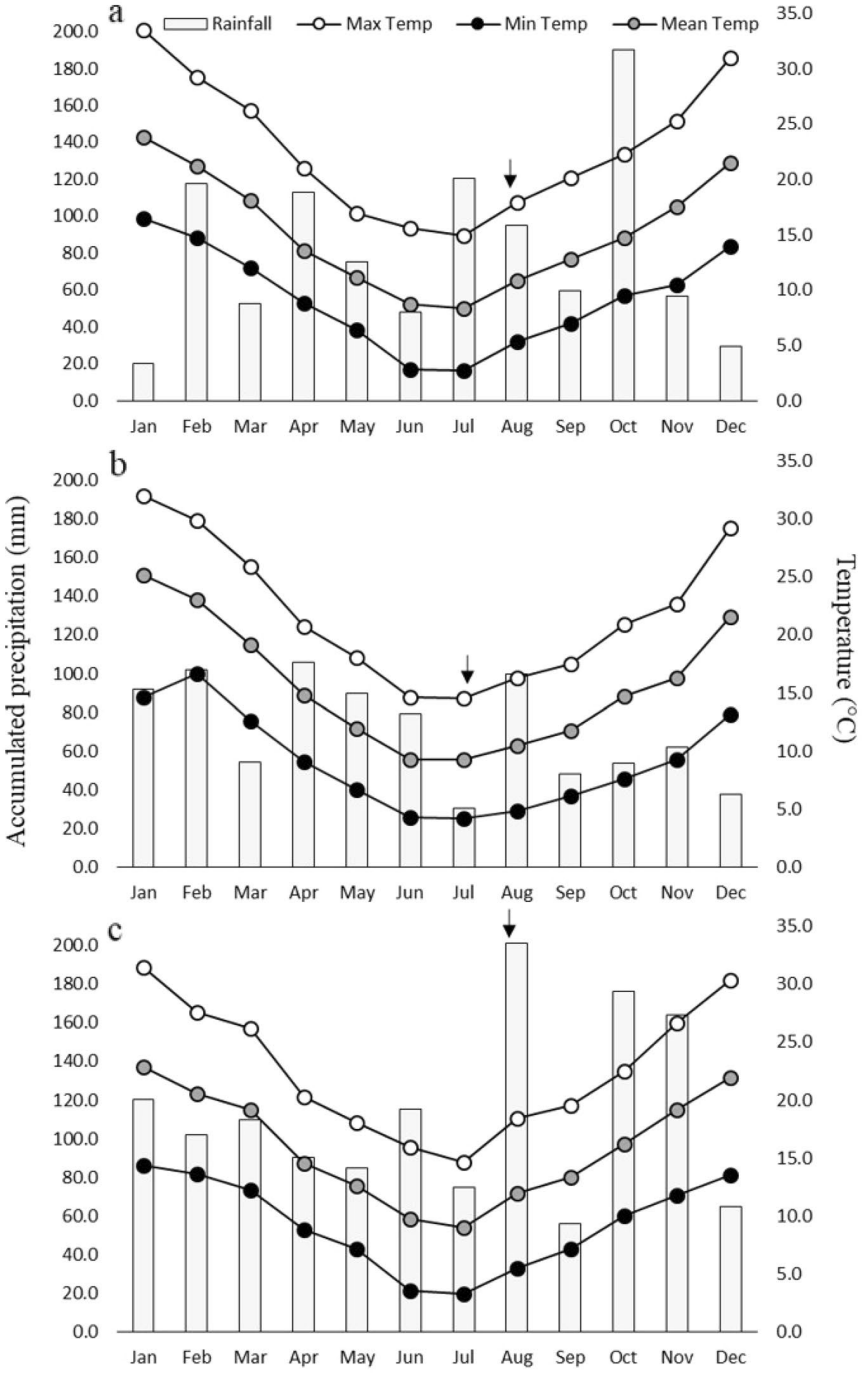
Accumulated precipitation and maximum, minimum and mean temperatures in three experimental environments of the Buenos Aires Province, Argentina (**a** CA14, **b** BW17 and **c** PS14). Arrows indicate sowing dates.

The experimental sites showed an uneven distribution of rainfall along the year, with low temperatures at crop sowing that continuously increased until the end of the crop cycle (Fig. 2). Total rainfall during the crop cycle ranged from 302 to 662 mm, enough to avoid water stress conditions along the experiments. The accumulated precipitation and mean temperatures during the crop cycle were above the normal in the three experiments (Table S3), showing the highest differences in CA14 and PS14.

The Argentinian durum wheat genotypes showed a wide range of variation in all the studied traits. Significant differences between genotypes (*P* < 0.05) were detected for all traits (Table 3). The effect of the G × E interaction (*P* < 0.04) was significant for all traits, except for aerial biomass. A significant effect of the environment (*P* < 0.05) was observed for most of the traits, except for plant height, grain number per spike and grain number per spikelet. The minimum, mean and maximum values for all the traits in the three environments are summarized in Table 3, while the data for every single environment are shown in Table S4. The mean value of days to heading was of 76 days in CA14, 86 days in BW17 and 73 days in PS14, with a range of variation among genotypes of 67–83 (CA14), 76.5–96 (BW17) and 66.5–82 days (PS14). On average, grain yield ranged from 1395 to 5394 kg ha^−1^ in the three environments, whereas the mean values for each environment were 3120, 4310 and 3313 kg ha^−1^ for CA14, BW17 and PS14, respectively.

**Table 3:** Summary of mean, ranges, *F* value (for genotype effect) and the heritability for 12 agronomic traits assessed in a durum wheat collection in three environments in Argentina.

Trait	Mean	SE	Min	Max	*F* value	H^2^
Grain yield (kg ha^−1^)	3581	64.24	1395.3	5393.9	14.61***	0.8
Harvest index	0.37	0.005	0.19	0.51	5.84***	0.57
Aerial biomass (g)	7.47	0.15	3.91	13.58	1.43*	0.09
Grain protein content (%)	12.35	0.11	9.46	17.45	2.36***	0.41
Plant height (cm)	92.63	0.87	78.4	139.1	12.38***	0.71
Heading date (days)	78.28	0.54	66.5	96	85.95***	0.93
Thousand kernel weight (g)	39.43	0.59	23.95	56.57	9.4***	0.79
Grain number per plant	72.28	1.52	32	133.05	3.52***	0.44
Spikes per plant	2.23	0.03	1.25	3.85	1.79**	0.26
Grain number per spike	33.37	0.38	19.74	50.79	5.55***	0.61
Grain number per spikelet	2.03	0.02	1.17	3.06	6.03***	0.58
Spikelets per spike	16.46	0.1	13.48	20.31	5.27***	0.55

*SE* standard error, *H*^*2*^ broad sense heritability.

*P < 0.05; **P < 0.01; ***P < 0.001.

The heritability ranged between 0.09 for aerial biomass and 0.93 for heading date (Table 3). Moderate to low heritability was found for spike number per plant (0.26), grain protein content (0.41) and grain number per plant (0.44), while the remaining traits (except for aerial biomass) showed moderate to high heritability (Table 3).

Pearson’s correlation coefficients exhibited a wide range of values for the traits (Table 4). Grain yield showed significant negative correlations with plant height (*r* = − 0.59) (*P* < 0.001), grain protein content (*r* = − 0.58) (*P* < 0.001) and heading date (*r* = − 0.36) (*P* < 0.01). Harvest index showed the highest positive correlation with grain yield (*r* = 0.72) (*P* < 0.001), followed by grain number related-traits (*r* = 0.5) (*P* < 0.001). Grain number per plant showed a similar correlation with spikes per plant (*r* = 0.75) (*P* < 0.001) and grain number per spike (*r* = 0.7) (*P* < 0.001). An in-depth analysis of the components of grain number in the spike showed that this trait was more strongly associated with the grain number per spikelet (*r* = 0.91) (*P* < 0.001) than with the spikelets per spike (*r* = 0.12) (*P* > 0.05).

**Table 4:** Pearson’s correlation coefficients for 12 agronomic traits evaluated in 59 Argentinian durum genotypes in three environments.

	HI	BPP	GPC	PH	HD	TKW	GNP	SP	GNS	GNs	sS
YLD	0.72***	− 0.01	− 0.58***	− 0.59***	− 0.36**	− 0.02	0.47***	0.21	0.51***	0.54***	− 0.13
HI		− 0.12	− 0.63***	− 0.73***	− 0.69***	0.03	0.51***	0.23	0.54***	0.7***	− 0.44***
BPP			0.08	0.28*	0.19	− 0.24	0.59***	0.69***	0.14	0.06	0.22
GPC				0.5***	0.43***	− 0.09	− 0.31*	− 0.07	− 0.37**	− 0.47***	0.27*
PH					0.47***	0.06	− 0.41**	− 0.27*	− 0.37**	− 0.48***	0.3*
HD						− 0.22	− 0.2	− 0.13	− 0.16	− 0.41**	0.61***
TKW							− 0.6***	− 0.34**	− 0.55***	− 0.39**	− 0.35**
GNP								0.75***	0.7***	0.67***	0.03
SP									0.08	0.12	− 0.07
GNS										0.91***	0.12
GNs											− 0.3*

*YLD* grain yield, *HI* harvest index, *BPP* aerial biomass, *GPC* grain protein content, *PH* plant height, *HD* heading date, *TKW* thousand kernel weight, *GNP* grain number per plant, *SP* spikes per plant, *GNS* grain number per spike, *GNs* grain number per spikelet, *sS* spikelets per spike.

*P < 0.05; **P < 0.01; ***P < 0.001.

### Relationships between genotypic and phenotypic data

**Figure 3:**
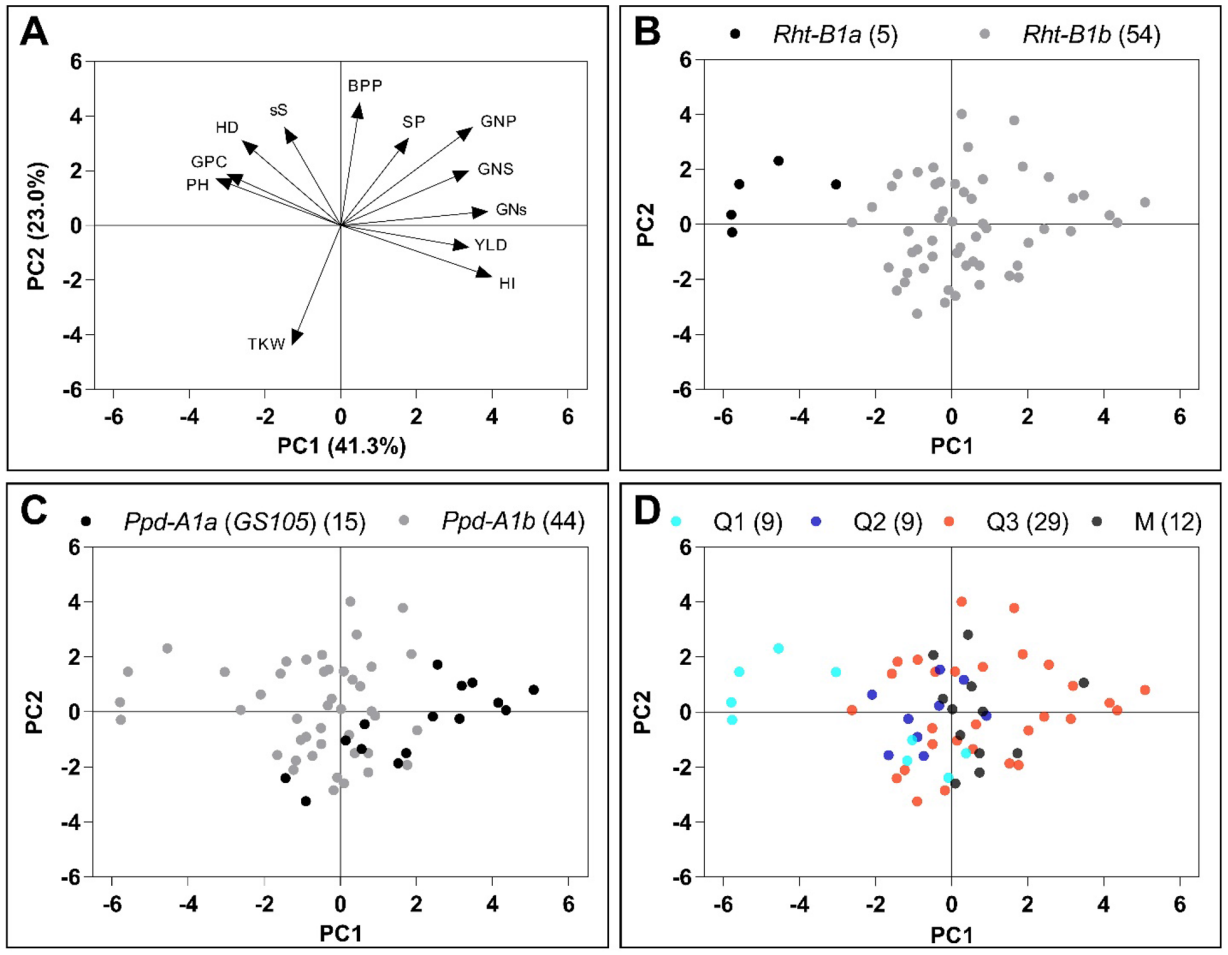
Principal components analysis (PCA) of agronomic traits for 59 Argentinian durum wheat genotypes. Traits correlation matrix is symbolized as vectors in A. Distribution of allelic variants at *Rht-B1* and *Ppd-A1* loci are shown in B and C, respectively. Colors represent the different groups from STRUCTURE in D. *YLD* grain yield, *HI* harvest index, *BPP* aerial biomass, *GPC* grain protein content, *PH* plant height, *HD* heading date, *TKW* thousand kernel weight, *GNP* grain number per plant, *GNS* grain number per spike, *GNs* grain number per spikelet, *sS* spikelets per spike, *SP* spikes per plant.

Principal Components Analysis (PCA) was used to illustrate the relationships between phenotypic variables and the different genetic groups. The first two components explained 64.3% of the total variation. PC1 explained 41.3% of the variation and it was mainly associated with positive values for harvest index, grain number-related traits and grain yield, and negative values for plant height and grain protein content (Fig. 3A). PC2 was mainly represented by negative values for thousand kernel weight and positive values for aerial biomass (Fig. 3A). PC1 divided the genotypes based on *Rht-B1* alleles (Fig. 3B). PC1 and PC2 were associated with negative and positive values for heading date, respectively, tending to separate the *Ppd-A1* allelic variants according to the two components (Fig. 3C). A clear relationship between the groups previously described (Q1–3 and M) and the PCA was not evident (Fig. 3D).

The effect of the allelic variants at the *Rht-B1* and *Ppd-A1* loci and the groups defined by STRUCTURE was evaluated for all the traits. The analysis of variance showed significant differences between the STRUCTURE groups in seven out of 12 traits (Table 5). Significant differences in grain yield were only observed for Q1, which showed a 30% reduction, on average, in grain yield relative to Q2, Q3 and M. The Q1 group also showed a lower harvest index and higher plant height and grain protein content (10.8, 13.5 and 5.9% on average, respectively) than the others. On the other hand, Q3 and the admixture groups showed higher grain number per spikelet (13.4%) and grain number per spike (12.6% on average) relative to Q1 and Q2 (Table 5).

**Table 5:** LSMeans for 12 agronomic traits considering the STRUCTURE groups and the allelic variants at the *Rht-B1* and *Ppd-A1* loci in three environments.

Trait	All	Q1	Q2	Q3	M	*Rht-B1a*	*Rht-B1b*	*Ppd-A1a* (*GS105*)	*Ppd-A1b*
Grain yield (kg ha^−1^)	3581	*2640 b*	*3744 a*	*3696 a*	*3887 a*	**2224 b**	**3707 a**	3815 a	3501 a
Harvest index	0.37	*0.33 b*	*0.36 a*	*0.37 a*	*0.38 a*	**0.29 b**	**0.37 a**	*0.4 a*	*0.36 b*
Aerial biomass (g)	7.46	7.46 a	7.51 a	7.45 a	7.43 a	8.00 a	7.41 a	7.49 a	7.45 a
Grain protein content (%)	12.3	*13.1 a*	*12.3 b*	*12.2 b*	*12.3 b*	**13.5 a**	**12.2 b**	*11.9 b*	*12.5 a*
Plant height (cm)	93	*104 a*	*95 b*	*89 c*	*91 bc*	**119 a**	**90 b**	*87 b*	*94 a*
Heading date (days)	78.3	80.2 a	79.5 a	77.3 a	78.3 a	**82.7 a**	**77.9 b**	*73.9 b*	*79.8 a*
Thousand kernel weight (g)	39.4	41.2 a	41.3 a	38.4 a	39.2 a	40.3 a	39.4 a	39.3 a	39.5 a
Grain number per plant	72.3	*59.8 b*	*68.8 ab*	*76.7 a*	*73.3 a*	**56.5 b**	**73.7 a**	*80.7 a*	*69.4 b*
Spikes per plant	2.23	2.07 a	2.28 a	2.67 a	2.23 a	**2.01 b**	**2.25 a**	*2.35 a*	*2.19 b*
Grain number per spike	33.4	*29.9 b*	*31.3 b*	*34.7 a*	*34.2 a*	**29 b**	**33.8 a**	*35.2 a*	*32.8 b*
Grain number per spikelet	2.03	*1.83 b*	*1.89 b*	*2.12 a*	*2.1 a*	**1.73 b**	**2.06 a**	*2.22 a*	*1.97 b*
Spikelets per spike	16.5	16.4 a	16.6 a	16.5 a	16.4 a	16.8 a	16.4 a	*15.9 b*	*16.7 a*

Different letters indicate significant differences at *P* < 0.05 according to Tukey’s test.

Significant values are in bold and italics.

The allelic variants at *Rht-B1* and *Ppd-A1* showed significant differences in nine out of 12 evaluated traits (Table 5). The genotypes carrying the semi-dwarfing *Rht-B1b* allele had an increase in grain yield of 1483 kg ha^−1^ (*P* < 0.001) relative to the genotypes carrying the *Rht-B1a*, whereas the genotypes with the *Ppd-A1a* allele (conferring photoperiod insensitivity) showed increases of 314 kg ha^−1^ relative to the ones carrying the *Ppd-A1b*, but this was not significant (*P* = 0.063) (Table 5). Moreover, genotypes carrying the *Rht-B1b* or the *Ppd-A1a* alleles had on average a shorter time to heading (4.8 or 5.9 days earlier, respectively) than those carrying the other allele (Table 5). In both cases, genotypes carrying the *Rht-B1b* or the *Ppd-A1a* alleles also showed significant reductions in plant height and grain protein content, and significant increases in harvest index, spikes per plant and grain number-related traits relative to the genotypes carrying the other alleles (*Rht-B1a* or *Ppd-A1b*) (Table 5). Furthermore, the genotypes with different alleles at *Ppd-A1* significantly differed in the number of spikelets per spike. No significant differences were found in the aerial biomass and thousand kernel weight for either STRUCTURE groups or for the allelic variants (Table 5).

As all the tall genotypes carried the *Ppd-A1b* variant, the genotypes carrying *Rht-B1b* or *Ppd-A1a* were grouped in order to study the effect of the two major genes separately. When the effect of *Ppd-A1* was only analyzed in the semi-dwarf genotypes (n = 54), significant differences persisted in seven out of nine traits (the spikes per plant and grain number per spike showed no significant differences). The effect of *Rht-B1* on photoperiod sensitivity genotypes (n = 44) was also tested and the significant differences persisted in eight out of nine traits (spikes per plant showed no significant differences). These results are summarized in Table S5.

## Discussion

Genetic diversity and allelic variants of major plant height and phenology genes had been studied in bread and durum wheat collections from various geographic regions, however not in the Argentinian durum wheat. Moreover, the relationships between genetic variability and agronomic traits had been poorly tested in durum wheat. In the current study, the population structure and the genetic variation at *Rht-B1* and *Ppd-A1* genes and its effects on agronomic traits were analyzed in a representative collection of Argentinian spring durum wheats.

Different allelic variants at the *Rht-B1* and *Ppd-A1* loci were found in our collection. The *Rht-B1b* semi-dwarfing allele had the highest frequency (92%) due to the rapid incorporation of this allele in Argentinian breeding programs since the 1970s, as reported by Rajaram et al.^2^. Regarding the *Ppd-A1* gene, two (*Ppd-A1b* and *GS105*) out of three main allelic variants reported in durum wheat^24^ were present in our collection, the photoperiod insensitivity *GS100* allele being absent. A fourth variant of this gene conferring photoperiod sensitivity was recently identified in durum wheat, named *Deletion Capelle-Desprez* (*DelCD*), which was found with a frequency of 20% in Mediterranean landraces and was not found in any of the 20 modern cultivars analyzed by Royo et al.^32^. This last variant was not analyzed here.

Our results showed that the photoperiod sensitive allele (*Ppd-A1b*) was more common in the Argentinian germplasm (75%) than the insensitivity one (*GS105*). On the contrary, higher frequency of the photoperiod insensitivity alleles at *Ppd-A1* than at *Ppd-A1b* had been found in other studies, with frequencies of 54% of *GS105* type allele and 10% of *GS100* in a collection of 474 durum wheat genotypes^33^. Royo et al.^32^ also observed a high frequency of the *GS105* allele (65%) followed by *GS100* (20%) in 20 Mediterranean adapted modern cultivars. Photoperiod insensitivity is considered one of the main tools to reach a crop wide adaptation^34^, which has been selected by breeders to improve the adaptation of the new varieties to more favorable conditions during grain filling^23^. The shuttle-breeding strategy takes advantage of using the insensitive alleles, which speeds up the rate of progress in wheat breeding^34^. Of the photoperiod insensitivity variants at *Ppd-A1*, the *GS100* allele showed a stronger effect on the reduction in flowering time in durum wheat than the *GS105*^23,24,35^. However, this allele was absent in Argentinian germplasm and it has only been found in a few durum wheat cultivars from Spain, Italy, North America and North Africa^32,33^. Therefore, the incorporation of the *GS100* allele is an interesting option to exploit novel sources of earliness in Argentinian durum wheat breeding programs.

The analysis of the genetic structure in our collection revealed a first main division between old, most carrying the *Rht-B1a* allele, and modern Argentinian genotypes. Subsequently, this analysis made it possible to divide the modern genotypes into two subpopulations which in general agreed well with the pedigree information. These results are in line with previous population studies using 125 AFLP and 56 SNP markers in a larger panel of 119 durum wheat accessions, which included the collection analyzed here^36^. This previous study indicated that the European and CIMMYT germplasms are the main genetic sources of the Argentinian durum wheat. The Q1 group, which included most of the oldest genotypes and Q2, conformed of modern genotypes, had the highest influence of the European Mediterranean germplasm based on its pedigree, mainly from Italy. The historical Italian cultivar ‘Cappelli’ and the landrace ‘Taganrog’ were mainly present in the Q1 pedigree. The genotypes belonging to the Q3 group, a second modern germplasm subset, exhibited major influence from the CIMMYT germplasm in its pedigree. Moreover, these differences are supported by the pairwise genetic differentiation between Q1 and the other two groups (mean *Fst* = 0.33). The moderate genetic differentiation estimated between the two modern germplasm groups, Q2 and Q3 (*Fst* = 0.19), could be attributed to their pedigrees. However, only 19.7% of the variability was explained for the differences between the groups. The highest variability was found within populations (80.3%), which was similar to the results shown in previous population analyses of durum wheat collections^26,27,37^.

This study demonstrated that ‘Bonaerense Quilaco’, registered in 1987, was the first cultivar released in Argentina carrying the photoperiod insensitivity allele (*GS105*). This allele was mainly present among genotypes belonging to Q3, the group with a high influence from CIMMYT in its pedigree. This result is in line with prior knowledge that the photoperiod insensitivity was derived from CIMMYT germplasm and then used in many breeding programs worldwide^20^.

The genetic diversity using 3565 SNP in our collection (*He* value of 0.33) showed similar or higher diversity values than those reported in other studies involving durum wheat collections^38–40^. Similar values of genetic diversity (0.30 on average) were found in a collection of 91 durum wheat landraces from Central Fertile Crescent by Baloch et al.^41^, whereas lower genetic diversity (0.19 and 0.12 on average) using SNP markers was detected in two different worldwide durum wheat collections of 150 and 370 accessions^26,27^.

The Q1 group showed differences in grain yield and several yield related traits. The accessions in this group reached a 30% lower yield in respect to the other groups. The increases in grain yield in modern genotypes belonging to the other groups (Q2, Q3 and M) can be partially attributed to the consistent reduction in plant height as a result of the introduction of the *Rht-B1b* semi-dwarfing allele. The pairwise correlations between the evaluated traits indicated that the harvest index was the main attribute that explained grain yield. Semi-dwarfism has been widely associated with increases in grain yield and harvest index due to a greater partition of assimilates towards plant reproductive structures in bread^42–44^ and durum wheat^45^. In our study, genotypes carrying *Rht-B1b* were 29 cm shorter than lines carrying *Rht-B1a*. Similar or higher reductions in plant height associated with semi-dwarfism were reported by McClung et al.^12^ and Miedaner et al.^46^. Moreover, the presence of *Rht-B1b* was associated with 10% reductions in grain protein content, a trait strongly, negatively correlated with yield. Previous studies have reported that semi-dwarfism is associated with similar reductions in grain protein content^47,48^. The lower grain protein content is caused by a dilution effect of the protein in the grains associated with high grain yield in semi-dwarfing genotypes^12^.

Of the two main yield components, the number of grains per plant was affected by different allelic variants for both *Rht-B1* and *Ppd-A1*, while no differences were found in the thousand kernel weight. The genotypes carrying the *Rht-B1b* or *Ppd-A1a* alleles showed 30 and 16% more grains per plant, respectively, than genotypes carrying the other allele. These increases were associated with increases in both the grain number per spike and the number of spikes per plant. The increases in grain number in the photoperiod insensitive genotypes could be attributed to the lower temperatures during the spike growth phase, as reported by Ratjen et al.^49^. Increases in grain number per spike and spikes per plant in semi-dwarf genotypes have previously been reported in durum wheat^12^. Detailed analysis of grain number per spike indicated that the differences between the *Rht-B1* and *Ppd-A1* allelic variants were explained more by the grain number per spikelet than by the spikelets per spike, as was shown by the correlation analyses between these last three traits. The presence of the *Rht-B1b* allele was associated with 15.6% more grains per spikelet than *Rht-B1a*, whereas no effect was observed between the alleles in the spikelets per spike. Álvaro et al.^50^ found similar increases in grains per spikelet in Italian and Spanish durum wheat varieties (10.5% and 15.8%, respectively) related to *Rht-B1b* and neither of them observed an effect of spikelets per spike associated with *Rht-B1* in these varieties. On the other hand, increases in grain number per spike caused by *Ppd-A1a* were mainly associated with increases in grain number per spikelet (13%), and also with low spikelets per spike (5%). On the contrary, Royo et al.^51^ and Arjona et al.^35^ reported that the number of spikelets per spike explained most of the differences in grain number per spike but it was associated with *Ppd-B1* variants, suggesting that *Ppd-A1* and *Ppd-B1* could be affecting the number of grains per spike through different mechanisms.

The Q3 and admixture groups included genotypes with a high grain number per spike, mostly due to increases in grain number per spikelet. In these groups, the *Ppd-A1a* allele was present in 43% and 17% of genotypes (in Q3 and admixture, respectively), but it was absent in the Q1 and Q2 groups. The *Ppd-A1a* allele has shown a positive effect on grain number related-traits. Thus, the increases in these traits in the Q3 and admixture groups could be associated with the effect of the *Ppd-A1* locus. However, the increase in grain number was not translated into higher yield within these groups, probably due to the influence of the negative correlation with the thousand kernel weight. Although the thousand kernel weight did not show any significant differences between the STRUCTURE groups or allelic variants, the Q3 and admixture showed lower values (6% on average) than Q1 and Q2.

Differences in the heading date between allelic variants were observed for both *Rht-B1* and *Ppd-A1*. Genotypes carrying the *Rht-B1b* or *GS105* (*Ppd-A1a*) allelic variants showed heading 4.8 and 5.9 days earlier, respectively. The effect of *Ppd-A1* variants on heading date showed similar patterns considering all genotypes or only the semi-dwarf ones. By considering only the *Rht-B1* alleles in the photoperiod sensitive genotypes, the differences in heading date were reduced to 3.3 days. A similar or lower effect in flowering time caused by *Rht-B1b* has previously been demonstrated in bread wheat^52,53^. Reductions in flowering time due to *Ppd-A1a* (*GS105*) compared with the *Ppd-A1b* allele were also reported previously, decreasing by 3–12 days in spring durum wheats in Spain and Mexico environments^23^. The early-flowering alleles tend to be associated with higher yields than the later-flowering ones, although in our study no-significant effect on grain yield associated with *Ppd-A1* was found, similar to the results of Arjona et al.^35^. However, several traits strongly correlated with yield showed differences between the *Ppd-A1* allelic variants. The *Ppd-A1a* (*GS105*) allele was associated with reductions of 7% in plant height and 5% in grain protein content, and increases in the harvest index with values of 0.40 related to the *GS105* and 0.36 to the *Ppd-A1b* alleles. Reductions of 11% in plant height and similar values of harvest index associated with these *Ppd-A1* variants were observed in 151 durum wheat landraces^51^.

Further studies in durum wheat collections like recombinant inbred lines (RILs), near isogenic lines (NILs) or mutants could provide more evidence on the effect of the alleles of *Rht-B1* and *Ppd-A1* loci on agronomic traits.

In conclusion, our results showed high genotypic and phenotypic variability available in the Argentinian germplasm of durum wheat, which can be exploited through breeding. Breeding efforts in Argentinian durum wheat fixed the *Rht-B1* semi-dwarfism allele in 1980 and introduced the *Ppd-A1* photoperiod insensitivity allele in modern cultivars after 1987. Our study showed that phenotypic variability in yield-related traits could be partially explained through the allelic variants of major genes and population groups. This highlights the potential to exploit this variability for a more targeted yield improvement considering the key role of using functional molecular markers in this approach.

## Methods

### Plant material

Fifty-nine Argentinian durum wheat (*Triticum turgidum* L. var. *durum*) genotypes were evaluated in the current study (Table 1), which were selected from the panel previously described by Roncallo et al.^36^. Our collection included 21 commercial durum wheat varieties released in the country between 1952 and 2015 and 37 advanced breeding lines developed by the Argentinian breeding programs, mostly since 1990. The landrace ‘Taganrong’, originally from Russia, which was one of the first founder genotypes of the Argentinian germplasm, was also included. The germplasm was obtained directly from the Argentinian breeding programs.

### Experimental design and growing conditions

Field trials were carried out in three environments in the southeast of Buenos Aires province, Argentina, considered to be representatives of the main durum wheat production area in Argentina. The trials were located at Cabildo [CA] (39° 36′ S, 61° 64′ W), Barrow [BW] (38° 20’S, 60° 13′ W) and Pieres [PS] (37° 46′ S, 58° 18′ W), and they were conducted during 2014 (CA and PS), and 2017 (BW). A randomized complete block design with two replications was used. The plots consisted of seven rows, 4.2 to 6.4-m long and 18 or 20 cm apart, but smaller areas of 5.5, 5.0 and 4.2 m^2^ were harvested in CA, BW and PS, respectively, to avoid border effects. All experiments were sown at 300 pl m^−2^. These trials were part of larger trials consisting of 170 genotypes in an alpha lattice design with two replications. The three experiments were conducted under rainfed conditions. Maximum, minimum and mean temperatures and accumulated precipitation were measured daily at weather stations located in or close to each experimental field (Fig. 2). Standard cultivation practices for each experimental field were adopted. Plots size details, soil texture and agronomic management (including fertilization and weeds and pest control) are shown in Tables S3 and S6.

### DNA extraction and genotypic characterization

DNA extraction was carried out from fresh leaves of 10-days-old seedlings of each genotype using a modified cetyltrimethylammonium bromide (CTAB) method as described in Dreisigacker et al.^54^. Our collection was genotyped using the 35 K Axiom Wheat Breeder’s Genotyping Array from Affymetrix^55^, with a call rate cutoff threshold ≥ 90%, performed at TraitGenetics (GmbH, Gatersleben, Germany). In addition, the allelic variation in the *Rht-B1* and *Ppd-A1* genes in the collection was assessed at CIMMYT using Kompetitive Allele‐Specific PCR (KASP) markers for polymorphisms described by Ellis et al.^56^ and Beales et al.^57^, respectively. The photoperiod sensitivity *Ppd-A1* gene was also evaluated using a sequence-tagged-sites (STS) marker and two KASP specific assays for *GS100* and *GS105* deletions^23,24^. The primers are summarized in Table S7.

The STS marker for *Ppd-A1* was assayed by PCR using two forward primers and a common reverse primer in a reaction mixture of 10 μl comprising final concentrations of 1X Buffer with Green Dye (Promega Corp., USA), 0.2 mM Deoxynucleoside 5’-triphosphates (dNTPs), 1.2 mM magnesium chloride, 0.5 μM of each primer, 1U of DNA polymerase (GoTaq®Flexi, Promega Corp., Cat. # M8295) and 50 ng of DNA. The cycling conditions were performed at 95 °C for 2 min, followed by 40 cycles of 94 °C for 1 min, 55 °C for 2 min, and finally 72 °C for 2 min. The amplified products were separated by electrophoresis in 12% polyacrylamide gel and visualized by a silver staining protocol.

A touchdown PCR protocol was used for KASP markers starting with 94° for 15 min, followed by 11 cycles of 94° for 30 s, 65°-55 °C for 60 s (−0.8 °C/cycle), 72 °C for 30 s and continued with 26 cycles of 94 °C for 30 s, followed by 57 °C for 60 s and a final step at 72 °C for 30 s. PCR was carried out arrayed in a 384 PCR plate, using 5 μl of PCR volume. DNA samples (150 ng/well) were briefly centrifuged and oven dried at 60 °C for 1 h. SNP-specific KASP reagents (5 μl), such as the assay mix and the 2X KASP Master mix, including the fluorescent dyes FAM and VIC, were added to dried DNA samples.

### Population structure and genetic diversity analyses

The population structure was analyzed with the software STRUCTURE 2.3.4^58^ using a model-based Bayesian approach assuming 1 to 10 groups (K) and 5000 burn-in iterations followed by 10,000 Markov Chain Monte Carlo (MCMC) iterations with five independent replicates for each K value. The Structure Harvester software^59^ implementing the Evanno method was applied to detect the true subpopulation (K) number^60^. Population structure was plotted using the Pophelper R library. Principal coordinate analysis (PCoA) was also performed in order to visualize the genetic relationships between the individuals and to compare this analysis with the groups defined by the STRUCTURE. The analysis of molecular variance (AMOVA) was used to estimate the percentage of variance explained between and within the STRUCTURE groups. The PCoA and the AMOVA were performed using the GenAlEx v6.5 software^61^.

Genetic diversity was assessed within each STRUCTURE group by calculating the percentage of polymorphic loci (%P), number of alleles (*Na*), number of effective alleles (*Ne*), Shannon information index (*I*) and the unbiased expected heterozygosity (*uHe* = (2N/(2N − 1)) * *He*; where N is the number of genotypes and *He* is the expected heterozygosity), and pairwise fixation index (*Fst*) using the GenAlEx v6.5 software.

### Phenotypic trait evaluations

Twelve traits were evaluated in all the 59 genotypes in the three environments. Grain yield was obtained as the weight of clean whole grains from the entire harvest plot, expressed as kg ha^−1^. Additional measurements were performed per plot: the heading date, as the number of days between sowing and when 50% of the spikes were in growth stage 55^62^; thousand-kernel weight calculated as the average weight (g) of three 100 grain samples; and the grain protein content (%) measured at INTA Barrow in a clean sample of 30 g of grains from each plot using Near-infrared spectroscopy (NIRs; FOSS®, Denmark), as an average of seven measurements, at 13.5% base humidity.

Ten plants were collected at random from the middle row of each plot at maturity and the plant height, aerial biomass, harvest index and the spike number from each plant were evaluated. Plan height (cm) was measured from the base to the top of the plant, including the awns. Aerial biomass (g) was recorded as dry weight of the aerial part of the plant. Harvest index was calculated as the ratio between the grain weight and aerial biomass from each plant. The spike number per plant was the number of fertile tillers per plant. Moreover, the number of spikelets and grains were counted for all the spikes in the 10 plants. The number of grains per plant was considered as the sum of the number of grains on all the spikes of the plant, and the grain number per spikelet as the ratio between grain number per spike and spikelet number per spike. Regarding the traits measured per spike, all spikes from each plant were considered and a mean value per plant was obtained. The mean values of the 10 plants for each trait were calculated and considered by plot.

### Statistical analysis

A linear mixed model with restricted maximum likelihood (REML) method (PROC MIXED; SAS University edition; SAS Institute, Inc., Cary, NC) was used to evaluate the effect of Genotype (G), Environment (E), and Genotype × Environment (G × E) interaction, considering these three factors as fixed and the effect of the block nested within environment as random. The broad sense heritability (H^2^) was estimated according to Nyquist^63^: H^2^ = σ^2^_G_/[σ^2^_G_ + σ^2^_GE_/E) + σ^2^_e_/rE)], where σ^2^_G_ is the genetic variance, σ^2^_GE_ is the genetic x environment interaction variance, σ^2^_e_ is the residual variance, E is the number of environments and r is the number of replications.

Best linear unbiased predictor (BLUP) was carried out using the REML method in SAS for estimating the phenotypic values for each trait and genotype in the three environments and these values were used for Pearson correlation coefficients (*r*) and principal components analyses (PCA).

The phenotypic differences were assessed for all the traits considering both the assignation to each STRUCTURE group and the allelic effect of the major genes using the REML approach in SAS. Each group was considered as a fixed effect and the G, the E, the G × E interaction and the block were nested within environment as random. Least-squares means (LSMeans) were calculated and, when the group effect was significant, the Tukey test at *P* < 0.05 was used.

### Ethics declarations

The plant material was provided by durum wheat Argentinian breeding programs, and sown in order to multiply and purify seed under the supervision of PFR. Research experiments follow the statements signed by Argentina under the International Convention for the Protection of new varieties of plants (UPOV, 1991, Art 15.1) and the Argentinian national law N° 20.247. In this study, permissions were not required to carry out field experiments in durum wheat as part of research activities.

## Supplementary Information

Supplementary Information.
