# Supplementary material for: Population structure, allelic variation at Rht-B1 and Ppd-A1 loci and its effects on agronomic traits in Argentinian durum wheat
Source: Sci Rep. 2022 Jun 10;12:9629. doi: 10.1038/s41598-022-13563-w (PMC9187632; doi:10.1038/s41598-022-13563-w)
Supplement: Supplementary file 1 — Supplementary Information. [file 41598_2022_13563_MOESM1_ESM.pdf]

Population structure, allelic variation at *Rht-B1* and *Ppd-A1* loci and its effects on agronomic traits in Argentinian durum wheat

Ana Laura Achilli[1], Pablo Federico Roncallo[1], Adelina Olga Larsen[2,3], Susanne Dreisigacker[4], Viviana Echenique[1,*]

[1] Centro de Recursos Naturales Renovables de la Zona Semiárida (CERZOS), Departamento de Agronomía, Universidad Nacional del Sur (UNS)-CONICET, Bahía Blanca, Argentina.

[2] Compañía Molinera del Sur, Bahía Blanca, Argentina

[3] Ex CEI Barrow, Instituto Nacional de Tecnología Agropecuaria (INTA), Tres Arroyos and CERZOS-UNS/CONICET, Bahía Blanca, Argentina

[4] International Maize and Wheat Improvement Center (CIMMYT), Mexico, DF, Mexico.

[*] Corresponding author. E-mail address: echeniq@criba.edu.ar (V. Echenique).

Table S1. Distribution of 3,565 SNP markers across the chromosomes and genomes of durum wheat.

**Chromosome**	**Total markers**
1A	200
1B	330
2A	225
2B	267
3A	181
3B	181
4A	130
4B	159
5A	175
5B	218
6A	176
6B	244
7A	233
7B	203
Unassigned	643
Total	3,565
Genome A	1320
Genome B	1602

Chromosome assignment was done basis of durum wheat genome cv. Svevo[1].

Table S2. Analysis of molecular variance (AMOVA) between genotypes based on groups defined by STRUCTURE.

Source	df	SS	MS	Estimated variance	Variance (%)
Between groups	3	26055	8685	503.1	19.7
Within groups	55	112801	2051	2050.9	80.3

Table S3. Summary of mean and range values for 12 agronomic traits of durum wheat collection in each environment. Cabildo 2014 (CA14), Barrow 2017 (BW17), Pieres 2014 (PS14), grain yield (YLD); harvest index (HI); aerial biomass (BPP); grain protein content (GPC); plant height (PH); heading date (HD); thousand kernel weight (TKW); grain number per plant (GNP); spikes per plant (SP); grain number per spike (GNS); grain number per spikelet (GNs); spikelets per spike (sS).

**Trait**	**CA14**			**BW17**			**PS14**		
	Mean	Min	Max	Mean	Min	Max	Mean	Min	Max
YLD	3120.2	1395.3	4160.1	4310.3	2606.1	5393.9	3312.6	1845.2	4797.6
HI	0.34	0.19	0.43	0.42	0.28	0.51	0.33	0.25	0.4
BPP	6.53	4.62	9.7	9.63	6.82	13.58	6.23	3.91	11.19
GPC	13.53	10.16	17.45	11.23	9.46	13.44	12.27	10.84	13.59
PH	91.47	79.45	134.88	94.49	78.4	139.1	91.95	79.9	120.2
HD	75.86	67	83	86.34	76.5	96	72.64	66.5	82
TKW	36.79	27.78	46.57	48.01	33.53	56.57	33.5	23.95	40.67
GNP	64.7	32	108.95	84.51	51.55	133.05	67.63	35.75	131.5
SP	2.08	1.25	2.9	2.46	1.8	3.85	2.16	1.25	3.6
GNS	32.32	19.74	47.45	35.75	25.54	50.79	32.06	23.45	42.85
GNs	2.03	1.17	3.06	2.05	1.55	2.93	2.03	1.42	2.65
sS	16.02	13.48	20.31	17.45	15.45	20.28	15.91	14.58	18.54

Table S4. LSMeans for 12 traits according to *Rht-B1* and *Ppd-A1* allelic variants considering the genotypes carried *Ppd-A1b* and *Rht-B1b*, respectively across three environments.

Trait	*Ppd-A1b* (n = 44)		*Rht-B1b* (n = 54)	
	Rht-B1a (n = 5)	*Rht-B1b* (n = 39)	*Ppd-A1a* (n =15)	*Ppd-A1b* (n =39)
Grain yield (kg ha^{-1})	2223.6 b	3665.1 a	3815.0 a	3665.1 a
Harvest index	0.29 b	0.36 a	0.40 a	0.36 b
Aerial biomass (g)	8.0 a	7.4 a	7.5 a	7.4 a
Grain protein content (%)	13.5 a	12.4 b	11.9 b	12.4 a
Plant height (cm)	119.4 a	91.2 b	87.4 b	91.2 a
Heading date (days)	82.7 a	79.4 b	73.9 b	79.4 a
Thousand kernel weight (g)	40.3 a	39.4 a	39.3 a	39.4 a
Grain number per plant	56.5 b	80.0 a	80.7 a	71.0 b
Spikes per plant	2.0 a	2.2 a	2.3 a	2.2 a
Grain number per spike	29.0 b	33.2 a	35.2 a	33.2 a
Grain number per spikelet	1.7 b	2.0 a	2.2 a	2.0 b
Spikelets per spike	16.8 a	16.6 a	15.9 b	16.6 a

Different letters indicate significant differences at $P < 0.05$.

Table S5. Sowing and harvest date, plot details and meteorological data for the three field trials.

Field trial	Sowing date	Harvest date	Plot size	Harvest plot area	Average Temperature (°C)		Available Accumulated water (mm)	
					Crop cycle	Long term[1]	Crop cycle	Long term[2]
CA14	11 August 2014	27 December 2014	6 m long and 1.4 m wide (7 rows, 0.2 m apart)	5.5 m^2	15.49	14.28	431	245
BW17	22 July 2017	29 December 2017	6.4 m long and 1.26 m wide (7 rows, 0.18 m apart)	5 m^2	14.98	13.74	302	245
PS14	6 August 2014	6 January 2015	4.2 m long and 1.4 m wide (7 rows, 0.2 m apart)	4.2 m^2	16.53	12.94	662	277

The average temperature and the available accumulated water were considered for the months of August to December. Data bases for long term: [1]https://es.climate-data.org/ [2]https://www.meteoblue.com/

Table S6. Soil texture, fertilization and weeds and pest control for the three field trials. No fungicides were applied in CA14 and PS14.

Field trial	Soil texture	Fertilization		Weeds control	Fungicide
		Sowing	Tillering	Tillering	
CA14	Sandy-loam	PDA (100 $kg.ha^{-1}$)	Urea (100 $kg.ha^{-1}$)	Axial (700 $cm^3.ha^{-1}$) + 2,4D (500 $cm^3.ha^{-1}$) + dicamba (150 $cm^3.ha^{-1}$)	-
BW17	Clay-loam	MicroEssentials S9 (200 $kg.ha^{-1}$)	Urea (210 $kg.ha^{-1}$)	Merit (6.5 $g.ha^{-1}$ + 100 $cm^3.ha^{-1}$)	Cripton X Pro (700 $cc.ha^{-1}$)
PS14	Clay-loam	PDA (150 $kg.ha^{-1}$)	Urea (380 $kg.ha^{-1}$; in two applications)	Starane + Metsulfuron (commercial dose), Foxtrop (1 $L.ha^{-1}$)	-

Table S7. Molecular marker summary.

Gene	Marker type	Marker name	Primer FAM / F1	Primer VIC / F2	Primer common / R	References
Rht-B1	KASP	wMAS000001	CCCATGGCCATCTCSAGCTG	CCCATGGCCATCTCSAGCTA	TCGGGTACAAGGTGCGGGCG	Ellis et al. (2002)
Ppd-A1	STS	no code	GTATGCGATTCGCCTGAAGT	CGTCACCCATGCACTCTGTT	CTGGCTCCAAGAGGAAACAC	Wilhelm et. al. (2009)
Ppd-A1 (*GS100*)	KASP	wMAS000030	CCAGTATCTTTAGATGCACCATGC	GCCGGCGGCTAAAAGG	CTATACAATGCTAAAGTCGCACAT	Wilhelm et. al. (2009)
Ppd-A1 (*GS105*)	KASP	wMAS000031	GGGGACCAAATACCGCTCG	CGTTTGGTGGTGGACGGG	GAAACAGAGGGGTGGTTTGAAAT	Wilhelm et. al. (2009)

References

1. Maccaferri, M. et al. Durum wheat genome reveals past domestication signatures and future improvement targets. *Nat. Genet.* **51,** 885–895 (2019).
